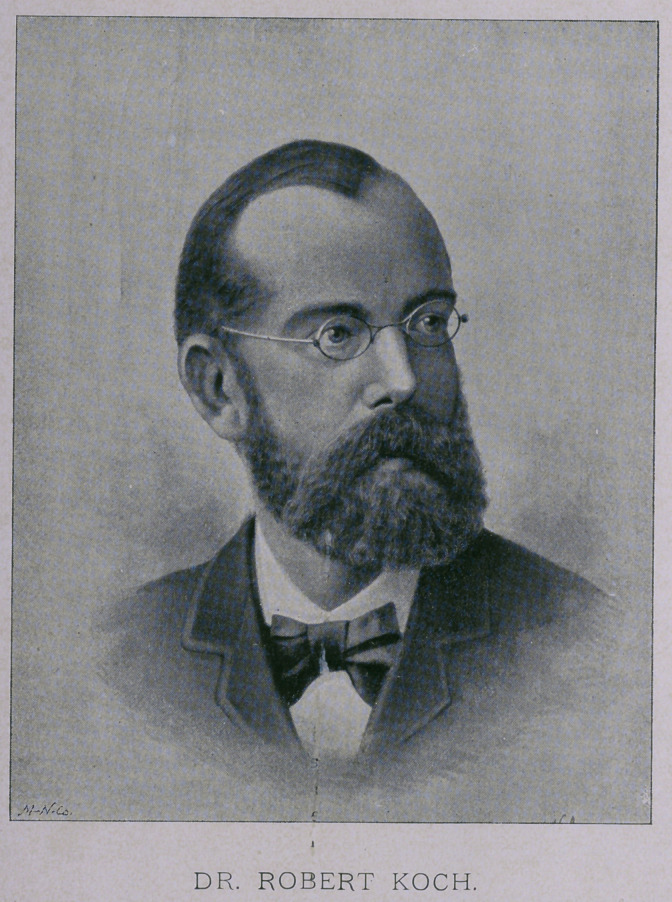# Koch’s Discovery

**Published:** 1890-12

**Authors:** 


[*From the "Buffalo Illustrated Express," November 23, 1890.*]

DR. ROBERT KOCH.

BUFFALO MEDICAL AND SURGICAL JOURNAL

A MONTHLY REVIEW OF MEDICINE AND SURGERY.

*EDITORS:*

THOMAS LOTHROP, M. D. - - WM. WARREN POTTER, M. D.

All communications, whether of a literary or business character, should be addressed to the editors : 284 FRANKLIN STREET, BUFFALO, N. Y.

VOL. XXX. DECEMBER, 1890. No. 5

### Editorial.

### KOCH'S DISCOVERY.

THE announcement that Professor Koch, of Berlin, has been able to arrest and even destroy tubercular processes by means of the injection of some remedy, was received with joy by the whole world. Koch's great reputation and the complete belief in his ability in this line of study, have led many to accept as fully proven that which is, as yet, in the stage of experimentation. It was unfortunate that Koch made any statement concerning his studies in this direction until he could divulge complete information in connection with the subject. We are entitled to know the remedy, as well as the means of using it. This knowledge, of course, he expects to give us, but in the meantime it is wise to refrain from any opinion, even on theoretical grounds, as to the probability of the success of the method.

This has been called "The Iron Age of Positivism." We demand proof before we believe ; and in questions of the cure of disease, time to observe the results o[illegible]atment is absolutely essential before we can obtain the proof [illegible] seek. Already the statement is made that it will take fully a year of trial before a reliable opinion can be formed as to the curability of consumption, either in advanced or early stages, by the proposed method. While, therefore, we must wait before definite knowledge is possible, it is confidently to be hoped that Koch's discovery will be proven to be all that the most ardent could wish it to be. Then he will stand among the greatest benefactors of his race.

It is to be noted that much of the confusion that exists up to the present in this matter is due to the lay press. In the desire for news it is announced one day that the remedy is a success, and the next that it is a failure. It would be much better if, in cases of this nature, where the greatest skill is necessary in order to understand and draw correct conclusions, the press would wait for thorough information before giving doubtful questions such wide publicity.

Two other things occur to us: First, that patients should not rush to Berlin under the hope that cure is at hand. It cannot be long before the general profession will know what the remedy is and be able to use it and test it the world over. Then proper advice can be given concerning it. And second, the profession ought not to use the remedy until they know what it is. It is a cardinal principle of medicine that unknown things should not be used. It ought not to be departed from in this case, even under the sanction of such a mighty name as Koch's.

We hope that before long we shall have reliable information upon which to suggest something more definite than is now possible. It may be that before this reaches the eyes of our readers, a further announcement will be made on the authority of the distinguished professor that will materially modify any opinion that could now be intelligently offered; so, let us wait patiently additional bulletins from Koch's laboratory on this all-absorbing and transcendentally important subject.

As a matter of interest at this time, it will be observed that we print a phototype of Dr. Koch in this number of the JOURNAL. It is an excellent likeness of the great laboratory investigator, taken from a recent photograph, and for which we are indebted to the courtesy of the *Buffalo Illustrated Express.*

## Figures and Tables

**Figure f1:**